# MiR-145 expression and rare NOTCH1 variants in bicuspid aortic valve-associated aortopathy

**DOI:** 10.1371/journal.pone.0200205

**Published:** 2018-07-30

**Authors:** Evaldas Girdauskas, Johannes Petersen, Niklas Neumann, Martin Ungelenk, Ingo Kurth, Hermann Reichenspurner, Tanja Zeller

**Affiliations:** 1 Department of Cardiovascular Surgery, University Heart Center Hamburg, Hamburg, Germany; 2 German Center for Cardiovascular Research (DZHK), Partner Site Hamburg/Lübeck/Kiel, Hamburg, Germany; 3 Institute of Human Genetics, Friedrich-Schiller University Hospital Jena, Jena, Germany; 4 Institute of Human Genetics, University Hospital RWTH Aachen, Aachen, Germany; 5 Clinic for General and Interventional Cardiology, University Heart Center Hamburg, Hamburg, Germany; Harvard Medical School, UNITED STATES

## Abstract

MicroRNAs (miRNAs) may serve as elegant tool to improve risk stratification in bicuspid aortic valve (BAV)-associated aortopathy. However, the exact pathogenetic pathway by which miRNAs impact aortopathy progression is unknown. Herewith, we aimed to analyze the association between circulating miRNAs and rare variants of aortopathy-related genes. 63 BAV patients (mean age 47.3±11.3 years, 92% male) with a root dilatation phenotype, who underwent aortic valve+/-proximal aortic surgery at a single institution (mean post-AVR follow-up 10.3±6.9 years) were analyzed. A custom-made HaloPlex HS panel including 20 aortopathy-related genes was used for the genetic testing. miRNAs were extracted from whole blood and miRNA analysis was performed using miRNA-specific assay. Study endpoint was the association between circulating miRNAs and rare genetic variants in the aortopathy gene panel. The study cohort was divided into a subgroup with rare variants vs. a subgroup without rare variants based on the presence of rare variants in the respective genes (i.e., at least one variant present). The genetic analysis yielded n = 6 potentially and likely pathogenic rare variants within the *NOTCH1* gene as being the most common finding. Univariate analysis between blood miRNAs and *NOTCH1* variants revealed a significantly lower expression of miR-145 in the subgroup of patients with *NOTCH1* variants vs. those without *NOTCH1* variants (i.e., delta Ct 4.95±0.74 vs. delta Ct 5.57±0.78, p = 0.04). Our preliminary data demonstrate a significant association between blood miR-145 expression and the presence of rare *NOTCH1* variants. This association may be indicative of a specific pathogenetic pathway in the development of genetically-triggered bicuspid aortopathy.

## Introduction

Dilatation of the proximal aorta, so-called *bicuspid aortopathy*, is present in approximately one half of the bicuspid aortic valve (BAV) patients and might be associated with an increased rate of adverse aortic events (i.e., aortic dissection / rupture) [1]. Cross-sectional aortic diameter has been traditionally used in the decision-making process regarding need of aortic surgery [2]. However, this parameter is obviously of limited value to predict the risk of adverse aortic events [3,4]. Phenotypic heterogeneity in BAV disease has been increasingly recognized during the last decade [5,6] and recent efforts have been focused on the identification of markers of progressive aortopathy [7]. In particular, microRNAs (miRNAs) have been suggested to be of potential value when diagnosing a progressive aneurysmal disease [7,8].

Although miRNAs have already been shown to be associated with bicuspid aortopathy [9], the specific molecular mechanisms by which miRNAs impact the development of aortopathy are still poorly understood. Here, we aimed to examine the association between specific blood miRNAs and rare genetic variants in candidate genes known to be associated with aortopathy and BAV, and to investigate their association with the severity of aortopathy.

## Methods

### Study population

Patients with isolated / predominant BAV insufficiency and aortic root dilation (i.e., so-called BAV root phenotype) were considered for this study. Patients with aortic stenosis (i.e., mean transvalvular pressure gradient ≥ 20.0 mmHg) were excluded. These inclusion criteria resulted in a total of 63 patients who were available for this study.

All 63 patients underwent a cross-sectional telephone-based follow-up (mean post-AVR follow-up 10.3 ± 4.9 years (range 0–19 years). Subsequently, these patients received a written follow-up questionnaire and were asked to answer specific questions regarding their family history of BAV and aortopathy. Follow-up visits were scheduled and included non-invasive proximal aortic imaging (transthoracic echocardiography and aortic CT/MRI) as well as peripheral blood sampling.

The study was approved by the ethics committee of the Medical Association Thuringia, Nr. 23333/2014/146 and each individual patient gave a written informed consent. The data for this study will be made available upon request, Due to ethical restrictions, data cannot be de-identified and data sharing is not allowed by the ethics committee of the Medical Association Thuringia, as well as due to restrictions of the data protection regulations of the European Commission. The contact person for data access is Beatrix Mattes (b.mattes@uke.de), current data trustee of the University Heart Center, Hamburg.

### Definitions and measurements

BAV was suspected if 2-dimensional echocardiographic short-axis imaging of the aortic valve demonstrated the existence of only two commissures delimiting two aortic valve cusps. The final decision regarding the bicuspidality of aortic valve was made based on the intraoperative description of valve anatomy by the attending surgeon. Sievers’ classification was used when classifying the bicuspid aortic valves [10]. Aortic valve insufficiency was quantified using the published echocardiography guidelines [11].

Proximal aortic diameters were assessed by multiple echocardiographic measurements in the parasternal long-axis view. All patients with aortic root diameter of ≥ 40.0 mm, as diagnosed by transthoracic echocardiography, underwent preoperative computed tomography (CT) or magnetic resonance imaging (MRI) of the thoracic aorta and aortic dimensions were measured in accordance to previously published recommendations [12].

### Genetic analysis

Details of the genetic analyses performed has been described before [13]. Briefly, a total of 20 candidate genes associated with BAV aortopathies were included in a custom aortopathy gene panel: *ACTA2*, *AXIN1*, *ELN*, *FBN1*, *FGF8*, *FN1*, *GATA5*, *HOXA1*, *KCNJ2*, *MMP9*, *MYH11*, *NKX2-5*, *NOS3*, *NOTCH1*, *PDIA2*, *SMAD6*, *TGFB1*, *TGFB2*, *TGFBR1 and UFD1L*. This aortopathy gene panel was designed based on data from the literature reporting genetic abnormalities in patients with BAV aortopathy and included the genes that were reported most consistently [1, 6, 14, 15]. Reported genetic variants of the aortopathy gene panel include only those listed in the ExAC database with an allele frequency of <0.01 or those that were not listed at all. The pathogenicity of the variants on the aortopathy gene panel was assessed using *in silico* prediction tool analysis (PolyPhen, MutationTaster, SIFT and LRT). Predicted effects of all potentially or likely pathogenic variants on the protein level are presented in our previous manuscript (13). Depending on the presence of rare variants, the study cohort was divided into a subgroup of patients with and without *rare* variants based on the presence of rare variants within *the respective genes* (i.e. at least one variant present) and were named e.g. *NOTCH1*(+) variant and *NOTCH1*(-) variant subgroups. Information on rare NOTCH1 variants are provided in S1 File and Table A in S1 File.

### miRNA analysis

Blood samples for miRNA analysis were collected the day before surgery. Total RNA was isolated from frozen whole blood samples using the PAXgene Blood miRNA kit (Qiagen, Hilden, Germany) according to the manufactures recommendation. Purification was carried out automated on a QIAcube system (Qiagen, Hilden Germany). Concentration of isolated RNA was measured on a NanoDrop N1000 System (peqlab) and 10ng of isolated RNA was used for miRNA analyses.

miRNAs to be analyzed were selected from the literature based on their previously reported association with aortopathies [9,16]. The following miRNAs were assessed: ***miR-1*, *miR-17*, *miR-18a*, *miR-19a*, *miR-20a*, *miR-21*, *miR-29b*, *miR-106a*, *miR-133a*, *miR-143*, *miR-145***. For normalization, miR-16 was used.

cDNA synthesis and miRNA analysis were performed using the TaqMan Advanced miRNA cDNA Synthesis Kit (ThermoFisher Scientific) and miRNA-specific TaqMan Advanced miRNA assays (ThermoFisher Scientific) on a 7900 HT Real Time System and Ct-values were normalized to miR-16 by the formula 2^-(Ct[miRNA]-Ct[miR-16])^ for Ct<40. In the case Ct value were ≥40, miRNAs were considered as undetermined.

### Control group

To validate our findings, we included a control group of 50 tricuspid aortic valve (TAV) patients without aortopathy who underwent aortic valve surgery due to valvular stenosis or insufficiency during the same study period. Measurements of circulating miR-145 values as well as genetic analysis of rare NOTCH1 gene variants which were previously identified in the study group was performed in this control group (n = 50). miRNA analysis was performed as described for the BAV study group. For genetic analyses of NOTCH1 variants in the control group de novo genotyping of the following SNPs was performed using 5′ nuclease assays according to the manufacturers´ recommendations (TaqMan assay, Applied Biosystems, Darmstadt): rs138504021 (c.4028C>T), rs201779159 (c.5414T>C). For rs752919688 (c.1334C>T) no reliable assay, i.e. primers and probes, could be generated.

### Statistics

Categorical variables are presented as percentages and continuous variables are expressed as mean ± standard deviation (SD). All statistical analyses were performed with the IBM SPSS 22.0 software (IBM Corp, New York, USA). Delta CT values of analyzed miRNAs were compared for differences using unpaired two-tailed t-test. The correlation analyses were performed using Pearson correlation coefficient. All p values of < 0.05 were considered statistically significant.

Univariate analysis was performed to analyze the potential impact of demographic parameters (age, gender), presence of co-morbidities (diabetes, chronic pulmonary disease, smoking, chronic renal failure), and the presence of rare *NOTCH1* variants on the expression patterns of circulating miRNAs.

## Results

### Characteristics of study participants

Demographics and intraoperative variables are summarized in **Table 1**. Our study cohort consisted of young, almost exclusively male patients with BAV insufficiency and aortic root dilatation, which underwent an aortic valve with/without aortic root surgery.

**Table 1:** Demographics and intraoperative variables.

Variable	Study cohort (n = 63)
Mean age (years)	47.3± 11.3
Male	58 (92)
BSA* (m^2^)	1.99± 0.7
FA of aortopathy/ SCD**	8 (13)
NYHA† class III / IV	27 (43)
Aortic root (mm)	48.1 ± 9.3
Arterial hypertension	38 (60)
Diabetes	3 (5)
History of smoking	34 (55)
Peripheral arterial disease	0 (0)
COPD ‡	2 (4)
Aortic valve repair	13 (21)
Composite graft replacement	21 (33)
Isolated AVR surgery	29 (46)
CPB time (min) ‖	84.0 ± 13.1
Cross-clamp time (min)	52.0 ± 8.2
Mean prosthesis size (mm)	27.8± 1.3
Mechanical prosthesis	38 (60)

Data presented as numbers (%) or as mean ± SD; FA -family anamnesis

*—body surface area

**—sudden cardiac death

† - New York Heart Association class

‡ - chronic obstructive pulmonary disease

‖ -cardiopulmonary bypass.

### Detection and correlation analyses of microRNAs

**Fig 1:**
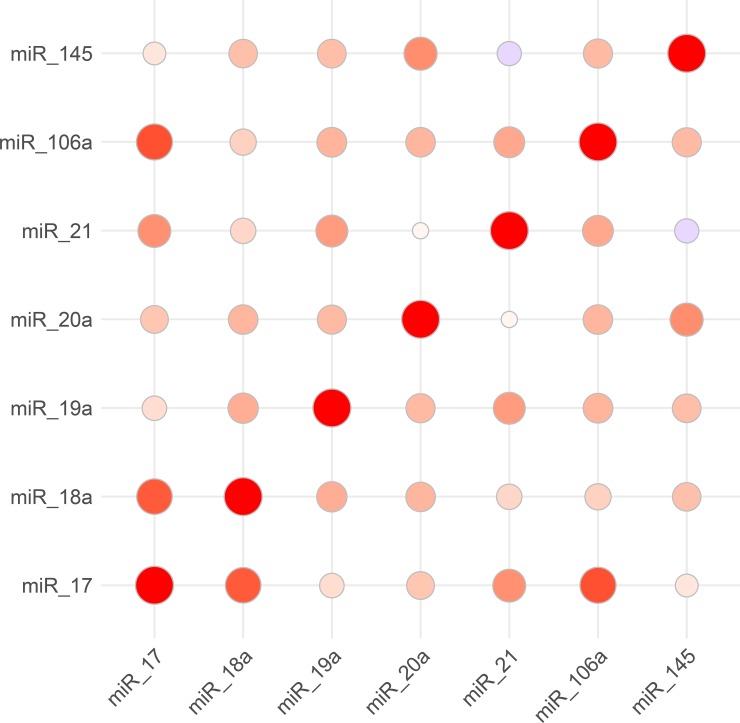
Correlation of individual microRNAs.

Four miRNAs (miR-1, miR-29b, miR-133a, and miR143) were undetectable. Results from the correlation analysis among the remaining miRNAs are presented in **Fig 1**. Although the values of most miRNAs correlated significantly, the strongest correlation was found between miR-17 and miR-106a (r = 0.84, p<0.001), miR-17 and miR-18a (r = 0.60, p<0.001) as well as between miR-145 and miR-20a (r = 0.58, p<0.001).

There was no significant association between the levels of the miRNAs and gender, different age profiles, and comorbidities. A significant inverse linear correlation was found between circulating miR-145 values and ascending aorta diameter (r = -0.25, p = 0.02).

### Genetic analysis and NOTCH1 variants

A total of 10 rare genetic variants in the *NOTCH1* gene were identified in our study cohort, with six of them classified as potentially and likely pathogenic based on *in-silico* prediction tool analysis **(Table 2).**

**Table 2:** Rare genetic variants within the 20 candidate genes in the aortopathy gene panel [13].

Gene	Rare variant
NOTCH1	c.1334C>T (p.T445M)*
	c.1862G>A (p.R621H)
	c.4492A>G (p.K1498E)
	c.4168C>A (p.P1390T)*
	c.4028C>T (p.A1343V)*
	c.5414T>C (p.L1805P)
AXIN1	c.1250G>A (p.R417H)
	c.2162C>T (p.A721V)
NOS3	c.3308T>A (p.V1103E)
	c.1263C>G (p.H421Q)
	c.502A>G (p.S168G)
ELN	c.515G>T (p.G172V)
	c.298G>A (p.A100T)
FN1	c.1808A>G (p.Q603R)
	c.3908T>C (p.V1303A)
FBN1	c.2956G>A (p.A986T)*
	c.3058A>G (p.T1020A)
GATA5	c.1159C>T (p.R387C)
MMP9	c.886G>A (p.G296S)*
NKX2-5	c.355G>T (p.A119S)*
FGF8	c.77C>T (p.P26L)*
PDIA2	c.583C>T (p.Q195X)
TGFBR1	c.437A>G (p.Y146C)

*- previously published pathogenic variants in association with clinical phenotype

All six *NOTCH1* gene missense variants resulted in a change of one amino acid in the *NOTCH1* protein structure. Five of the six *NOTCH1* variants are located within the extracellular domain. In fact, c.1334C>T (p.T445M), c.1862G>A (p.R621H), c.4028C>T (p.A1343V) and c.4168C>A (p.P1390T) affect epidermal growth factor (EGF)-like domains. Each EGF-like repeat is composed of approximately 40 amino acids, and its structure is defined largely by six conserved cysteine residues that form three conserved disulfide bonds. Three of the detected changes, c.1862G>A (p.R621H), c.4028C>T (p.A1343V), and c.4492A>G (p.K1498E) are in close proximity to one of the cysteines. The alteration of the amino acid sequence might also alter the capability to create disulfide bonds and thus impair ligand binding. In addition, each EGF-like repeat can be modified by O-linked glycans at specific sites. The c.1334C>T (p.T445M) variant at such a conserved site that is predicted to undergo glycosylation might well affect protein function [17]. The mutation c.4492A>G:(p.K1498E) is located in the LNR2 domain. The LNR (Lin-12/Notch repeat) region is a hallmark of the Notch receptor family and is involved in Notch signaling. An altered amino acid sequence might prevent the initiation of the ligand-induced proteolytic cleavage and release of the intracellular regulatory domain. The functional consequence of the intracellularly located c.5414T>C (p.L1805P) variant is difficult to predict, however, the incorporation of a proline may impact the secondary structure of the protein.

Clinical characteristics and severity of BAV-associated aortopathy were compared between the patients’ subgroup with identified *NOTCH1* gene variants (i.e., at least one potentially or likely pathogenic *NOTCH1* variant, n = 5) and the subgroup without pathogenic *NOTCH1* variants (n = 58). No clinically relevant differences could be identified between *NOTCH1*(+) variant and *NOTCH1*(-) variant subgroups in terms of age at presentation (i.e. 52.1 ± 11.4 years vs 47.3 ± 11.2 years, P = 0.4), pre-AVR aortic dimensions (i.e. 45.3 ± 12.1mm vs 48.1 ± 8.3, P = 0.4) and family history of BAV/aortopathy. Of note, all *NOTCH1* (+) patients had an isolated aortic valve regurgitation and dilation of the aortic root. Except for the raphe region, there were no major calcifications in the aortic cusps or severe stenotic component of the aortic valve lesion.

### Association between miRNA expression and NOTCH1 variants

**Fig 2:**
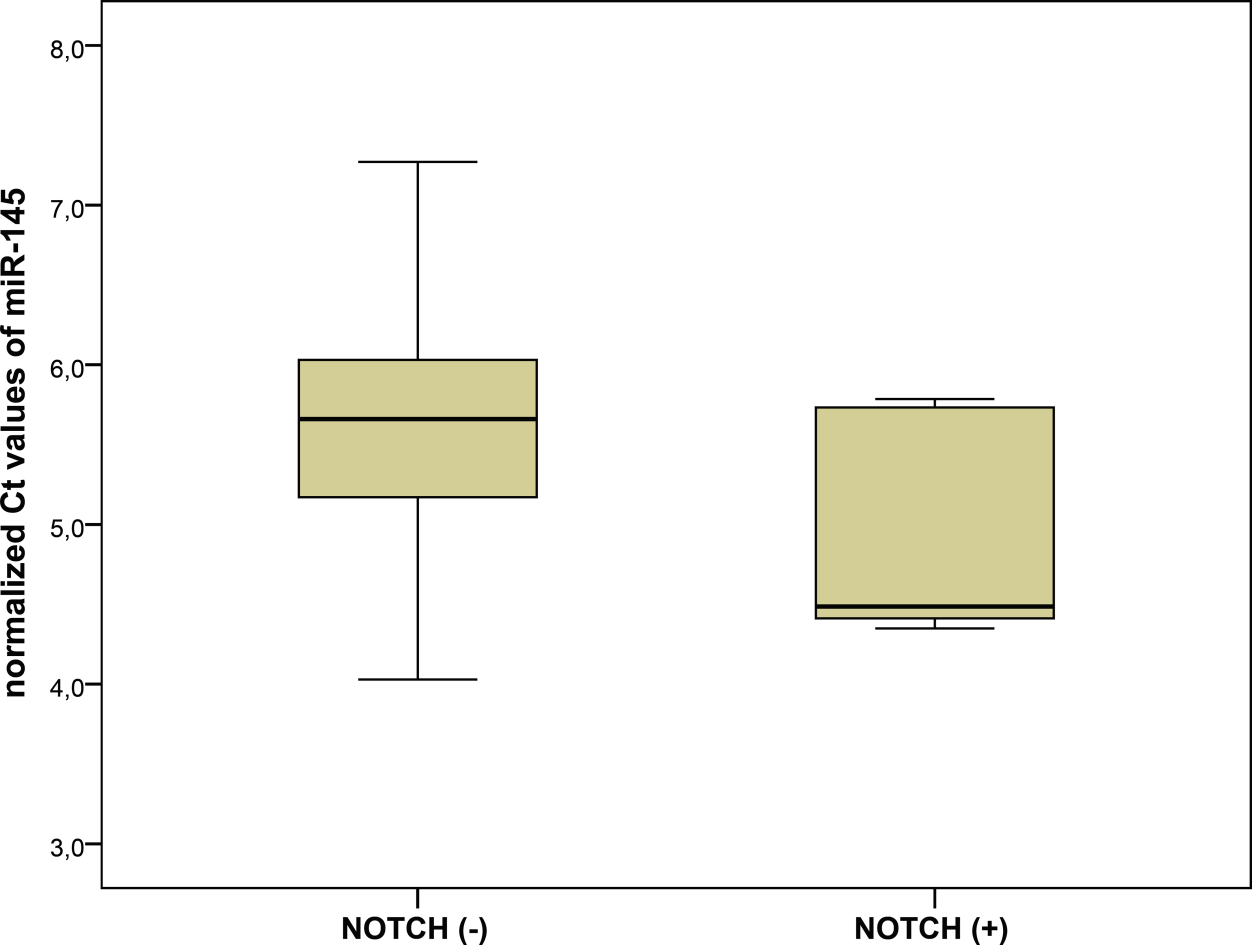
Expression of miR-145 in patients with and without rare *NOTCH1* variants.

We analyzed the association between the presence of *NOTCH1* variants and miRNA expression. A significantly lower expression of blood miR-145 was found in the subgroup with *NOTCH1* variants as compared to patients without *NOTCH1* variants (delta Ct 4.95±0.74 vs. delta Ct 5.57±0.78, p = 0.045) (**Table 3, Fig 2**). The remaining miRNAs demonstrated no significant differences in the expression patterns between *NOTCH* (+) and *NOTCH* (-) subgroups.

**Table 3:** Expression of microRNAs in patients with and without potentially/likely pathogenic NOTCH1 variants. Normalized delta Ct values are shown.

miR*	Whole cohort, (n = 63)	NOTCH1 (+) subgroup, (n = 5)	NOTCH1 (-) subgroup, (n = 58)	p value**
**miR-145**	5.52±0.79	4.95±0.74	5.57±0.78	0.045
**miR-21**	5.30±0.73	5.73±0.78	5.26±0.73	0.173
**miR-17**	1.91±0.65	2.23±0.73	1.88±0.65	0.254
**miR-19a**	6.26±1.46	5.74±0.78	6.30±1.17	0.291
**miR-20a**	5.08±1.13	4.63±1.17	5.12±1.13	0.360
**miR-18a**	7.12±1.46	5.81±3.29	7.24±1.18	0.388
**miR-106a**	5.77±0.52	5.86±0.35	5.76±0.53	0.673

*—delta CT values, normalized for miR-16 values

**- p value for comparison NOTCH1 (+) vs. NOTCH1 (-)

### miR-145 expression and the progression of aortic root diameter

To analyze the potential relation of miR-145 to the development of BAV aortopathy, we examined the association between expression of blood miRNAs and clinical markers of aortopathy progression.

The progression of aortic root diameter was assessed in the subgroup of 39 (46%) BAV root phenotype patients who did not receive an aortic root surgery (i.e., an isolated aortic valve replacement only). The mean length of MRI follow-up was 11.1 ± 5.5 years (a total of 312 patient-years). An MRI-defined progression of aortic root diameter ≥ 3.0 mm (i.e., measured at the level of the sinus of Valsalva) was revealed in 7 post-AVR patients with a range of progression being between 3.0–12.0 mm.

A significant association between miR-145 as well as miR-17/miR-106a and the progression of aortic disease was observed. The values of miR-145 (5.48±0.79 vs. 6.21±1.12, p = 0.04) and miR-17/miR-106a (1.63±0.42 vs. 2.11±0.63, p = 0.044) were significantly lower in patients who experienced aortopathy progression (i.e., diameter increase ≥ 3mm) vs. those with “stable” aortic diameters (i.e., diameter increase < 3mm) (**Table 4**). Of note, 4 of the 39 followed patients had pathogenic *NOTCH1* variants and all of them were male.

**Table 4:** Expression of microRNAs in patients with and without aortopathy progression. Normalized delta Ct values are shown.

MicroRNA*	Aortic surgery (-), (n = 39)	Progressive aortopathy, (n = 7)	Stable aortopathy, (n = 32)	p value**
**miR-106a**	5.84±0.44	5.48±0.37	5.92±0.42	0.023
**miR-145**	5.61±0.85	5.48±0.79	6.21±1.12	0.04
**miR-17**	2.04±0.63	1.63±0.42	2.11±0.63	0.044
**miR-19a**	6.22±0.96	5.81±0.62	6.28±1.02	0.295
**miR-20a**	5.06±1.21	5.30±0.87	4.92±1.25	0.479
**miR-21**	5.36±0.63	5.21±0.84	5.39±0.62	0.557
**miR-18a**	7.00±1.72	7.27±0.48	6.90±1.92	0.640

*—delta CT values, normalized for miR-16 values

**- p value for comparison progressive aortopathy vs. stable aortopathy

Furthermore, a correlation analysis between the levels of circulating miR-145 and the absolute aortic diameter in the subgroup of 39 patients who did not receive aortic surgery showed a significant moderate inverse correlation between these two variables (r = -0.48, p = 0.03).

### Comparison with TAV group

None of the rare NOTCH1 variants could be identified in the control group of TAV patients without aortopathy. Simultaneously, the expression of circulating miR-145 was significantly higher in the TAV control group vs. BAV study group (i.e., 25.9 ± 3.9 vs. 20.7 ± 0.6, p<0.001).

## Discussion

The primary aim of this project was to examine the association between specific miRNAs and rare genetic variants known to be related to aortopathy and to investigate the association of miRNAs and the severity of aortopathy.

Our main results show that i) miR-145 is associated with rare genetic variants within the *NOTCH* 1 gene and ii) that miR-145 correlates with the progression of bicuspid aortopathy during the follow up. While our previous publication [13] focused primarily on the whole spectrum of rare genetic variants in BAV patients, without addressing biomarkers and without analyzing the pathogenetic linkage between NOTCH1 variants, circulating miRNAs and aortopathy, our current manuscript aims to establish a potential pathogenetic pathway in the development of BAV aortopathy and therefore presents the subsequent and in-depth analysis of the previously identified rare genetic variants.

The pathogenesis of BAV-associated aortopathy is incompletely understood. Hemodynamic effects and primary genetic disorder have been proposed to interact to various degree, simultaneously causing the progression of BAV-associated aortopathy [1]. Traditional criteria based on the aortic size are insufficient to reliably stratify the complication risk in BAV aortopathy [3,4]. Therefore, circulating biomarkers and in particular miRNAs may be valuable tools to improve the prediction of aortic events in BAV disease [7–9]. However, the pathogenetic pathways by which miRNAs are involved in the development of aortopathy are still poorly understood [18,19].

Patients included in this study were BAV root phenotype patients who are known to have the most aggressive form of aortopathy and a predominantly genetically-triggered aortic disease [5,20]. Recent studies reported genetic abnormalities in *TGFBR2*, *FBN1*, *NOTCH1* and *SMAD2 genes* in this specific patient cohort [14], while the clinical data indicated an increased risk of life-threatening aortic events after an isolated aortic valve surgery [20] suggesting a relation between the molecular mechanisms linked to these genes and the severity of aortic events.

Of main interest is the finding of a significant association between rare variants in the *NOTCH1* gene and the expression of miR-145 and the significant association of miR-145 and aortopathy progression during the follow-up. *NOTCH1* signaling network is an evolutionary conserved intercellular signaling pathway that plays a role in a variety of developmental processes by controlling cell fate decisions. It is involved in the angiogenesis and negatively regulates endothelial cell proliferation and migration as well as the angiogenic sprouting.

Our findings are in accordance with previous studies showing that miRNAs are downregulated in progressive vasculopathies [16]. Specifically, miR-145 has previously been shown to influence vascular smooth muscle cell (VSMC) differentiation through post-transcriptional repression of proliferation-associated proteins and thereby has implications for vascular diseases [16,21]. Expression of miR-145 was significantly downregulated in the vascular walls with neointimal lesion formation and in cultured dedifferentiated VSMCs [21]. Based on these findings, miR-145 has been proposed as a novel phenotypic modulator of VSMCs [21].

Furthermore, Boucher et al. proposed a potential linkage between miR-145 expression and the *NOTCH* signaling [18]. The authors suggested a common signaling pathway that promotes the VSMC contractile phenotype, including an increased *NOTCH* signaling and simultaneous upregulation of miR-145 expression, and leads to an increased differentiation and decreased proliferation [18]. Conversely, the inhibition of basal *NOTCH* signaling is associated with decreased levels of miR-145 [18]. In accordance to these findings, we found a decreased miR-145 expression in the study subgroup with rare variants of *NOTCH1* gene. Rare *NOTCH1* gene variants, as identified in this study, may cause deleterious effects on the protein structure and therefore be responsible for attenuated basal *NOTCH* signaling which may be associated with the decreased miR-145 levels and resulting progression of aortopathy. Most of the detected *NOTCH1* variants are located within important ligand binding domains. Interfering with the capability to form disulfide bonds or disrupting proper post-translational modification like glycosylation, these mutations are likely to affect *NOTCH1* function. This is also supported by *in silico* prediction tools (**Table 2**).

The impact of the *NOTCH1* pathway in the pathogenesis of proximal aortic disease and BAV was reported in several studies [14,15,22]. Three *NOTCH1* variants identified in our study (i.e., *c*. *1334C>T (p*. *T445M)*, *c*. *4168C>A (p*. *P1390T)*, *c*. *4028C>T (p*. *A1343V)* were previously reported in thoracic aortic aneurysms in BAV patients [14,15], as well as in patients with Adams-Oliver syndrome which is associated with variable cardiac anomalies (i.e., ventricular septal defects, pulmonary atresia) [23]. Similar to the findings in the whole *NOTCH (+)* subgroup, there was a tendency towards lower miR-145 expression in the two BAV patients with the previously published *NOTCH1* variants (5.05±0.93 vs. 5.57±0.78, p = 0.25). Furthermore, none of these rare NOTCH1 variants could be identified in the control group of TAV patients without aortopathy. Corresponding to these findings, TAV patients had significantly higher miR-145 expression as compared to the BAV study group.

**Fig 3:**
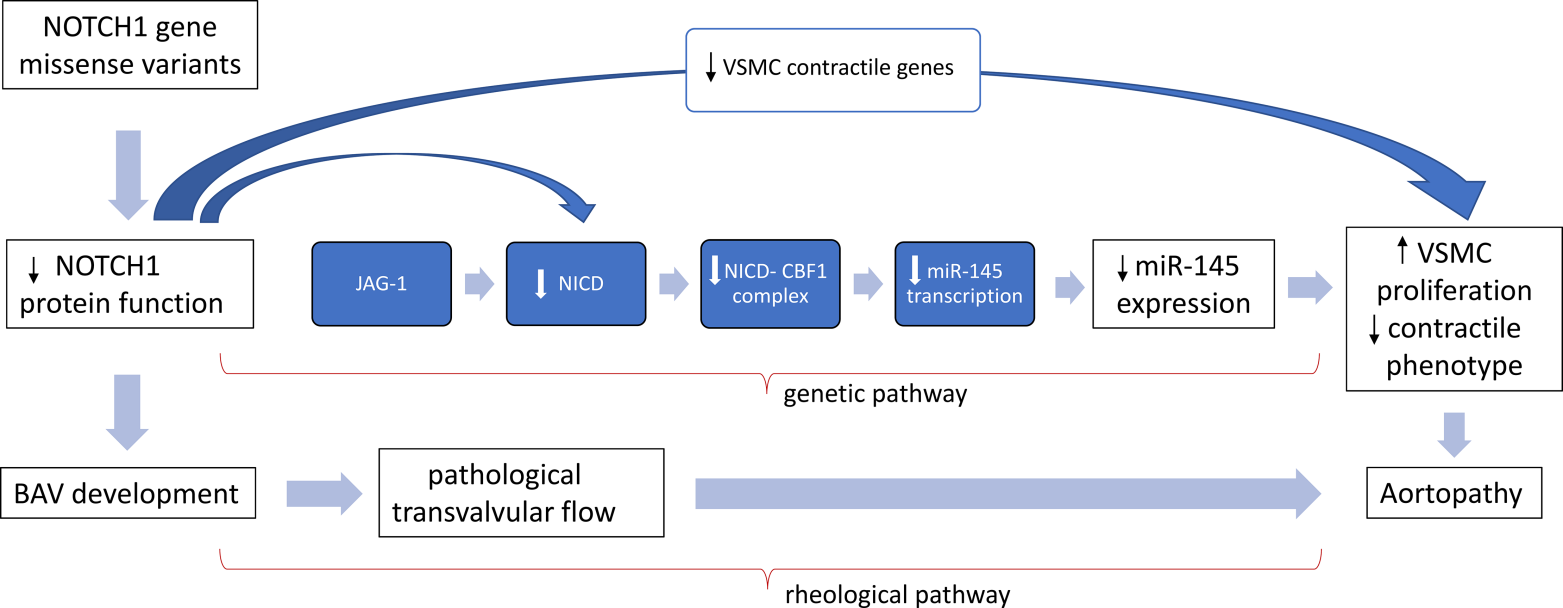
Potential mechanistic linkage between NOTCH1 variants, miR-145 expression and bicuspid aortopathy.

In summary, we provide preliminary data which supports the association the association between BAV aortopathy, rare NOTCH1 variants, and miR-145 expression. **Fig 3** displays the potential mechanistic link between NOTCH1 variants, miR-145 expression and bicuspid aortopathy. However, the final evidence for the proposed genetic pathway has yet to be demonstrated on the molecular basis.

Although our previous manuscript revealed a wide spectrum of genetic abnormalities in BAV root phenotype patients [13] we focused here primarily on rare *NOTCH1* variants which was because (a) *NOTCH1* variants had the highest frequency as compared to the genetic abnormalities in the remaining 11 candidate genes; (b) the previously reported potentially pathogenetic linkage between basal NOTCH1 signaling and miR-145 expression (**Fig 3**).

In addition, our study findings are in accordance with data published by Wu and co-authors and indicate that the expression of miR-17-associated miRNAs (a total of 9 miRNAs, including miR-17 and miR-106a) correlates with the severity of aortopathy [16]. Similar to the miR-145 expression, miR-17/miR-106a blood levels were significantly downregulated in BAV patients who experienced the progression of aortopathy during the follow-up.

### Study limitations

This study has some limitations. The present miRNAs analysis targeted 11 miRNAs, all previously reported in association with aortopathy [6,13] and 20 candidate genes known to be related to aortopathy. A more comprehensive analysis of the whole spectrum of human miRNAs and genetic variants, e.g. by sequencing technologies would have enabled us to reveal further miRNAs and gene variants potentially associated with the BAV root phenotype and to expand the list of molecular biomarkers and gene variants potentially relevant to the phenotype. In addition, the functional impact of genetic variants identified remains to be clarified. Furthermore, expression of miR-145 in the aortic tissue would be of great scientific interest and would potentially expand our findings. Unfortunately, our study cohort of BAV root phenotype was collected over a very long period and no aortic tissue is available in two thirds of patients.

Although we identified a statistical association between levels of circulating miR-145 and genetic variants in the NOTCH1 gene in the presented study, further analyses need to be performed to provide the direct link between the cellular expression of miR-145 and NOTCH1 variants.

This study included limited number of BAV patients and therefore needs to be seen as of preliminary character. We did not include a control group with tricuspid aortopathy and therefore cannot answer the question whether the differential expression of circulating miRNAs is specific for BAV aortopathy. Finally, our patients had their primary operation over a 20-year period, and most of them were in their 60s or 70s at the time of last follow-up. Their parents were not available for genetic analysis, and the data on siblings and children were insufficient. Therefore, we unable to get the full picture on the penetrance patterns of identified variants in the families or substantiate the role of *de novo* variants in the study cohort.

### Conclusions

Our preliminary data demonstrate a significant association between blood miR-145 expression and the presence of rare *NOTCH1* variants. This association may be indicative of a specific pathogenetic pathway in the development of genetically-triggered bicuspid aortopathy and highlights the role of specific miRNAs in the transmission of genetic defects inducing vasculopathy.

## Supporting information

S1 File (Table A) Rare NOTCH1 variants identified in the current study.
(DOCX)
