# Supplementary material for: MiR-145 expression and rare NOTCH1 variants in bicuspid aortic valve-associated aortopathy
Source: PLoS One. 2018 Jul 30;13(7):e0200205. doi: 10.1371/journal.pone.0200205 (PMC6066209; doi:10.1371/journal.pone.0200205)
Supplement: S1 File — (Table A) Rare NOTCH1 variants identified in the current study. (DOCX) [file pone.0200205.s001.docx]

**MiR-145 expression and rare NOTCH1 variants in bicuspid aortic valve-associated aortopathy**

Evaldas Girdauskas^1,2, *^, Johannes Petersen^1,2^, Niklas Neumann^1,2^, Martin Ungelenk^3^ , Ingo Kurth^4^, Hermann Reichenspurner^1,2^ PhD, Tanja Zeller^2,5^.

1- Department of Cardiovascular Surgery, University Heart Center Hamburg, Germany

2- German Center for Cardiovascular Research (DZHK), Partner Site Hamburg/Lübeck/Kiel, Hamburg, Germany

3- Institute of Human Genetics, Friedrich-Schiller University Hospital Jena, Jena, Germany

4- Institute of Human Genetics, University Hospital RWTH Aachen, Aachen, Germany

5- Clinic for General and Interventional Cardiology, University Heart Center Hamburg, Germany

***corresponding author**

**E**-mail: e.girdauskas@uke.de

**S1 File**. Supplementary Material. (Table A) Rare NOTCH1 variants identified in the current study

| **Gene** | **Rare variant** | **Allele frequency in** **ExAC** | **CADD phred score** | **GERP** | **Phenotype** |
| --- | --- | --- | --- | --- | --- |
| **NOTCH1 (NM_017617)** | c.1334C>T (p.T445M)* | 0.000008441 | 23.4 | 3.65 | VSD/AOS |
|  | c.1862G>A (p.R621H) | 0.001199 | 28.5 | 5.24 |  |
|  | c.4492A>G (p.K1498E) | 0.00004656 | 1.074 | 2.89 |  |
|  | c.4168C>A (p.P1390T)* | 0.0006212 | 14.78 | 3.65 | BAV + TAA |
|  | c.4028C>T (p.A1343V)* | 0.001881 | 23.2 | 4.66 | BAV + TAA |
|  | c.5414T>C (p.L1805P) | 0.00002519 | 28.9 | 4.61 |  |

* previously published mutation, in combination with congenital cardiovascular syndromes

AOS- Adams-Oliver syndrome; BAV- bicuspid aortic valve; TAA- thoracic aortic aneurysm; VSD- ventricular septal defect;

**CADD phred score** - “meta-annotation” tool that uses information from many functional annotation tools (SIFT, PolyPhen, etc). Scaled CADD score of 20 means that a variant is amongst the top 1% of deleterious variants in the human genome.

**ExAC-** the Exome Aggregation Consortium

**CADD and GERP scores**: the higher scores, the more deleterious SNPs;

**GERP -** Genomic Evolutionary Rate Profiling, is a method for producing estimates of evolutionary constraint of a specific site. Constraint intensity (most conserved site) at each individual alignment position is quantified in terms of a “rejected substitutions” (RS) score which ranges from -12.3 (least) to 6.17(most).

Anticipated consequences of rare NOTCH1 variants:

1. c.1334C>T (p.T445M), c.1862G>A (p.R621H), c.4028C>T (p.A1343V) and c.4168C>A (p.P1390T) affect epidermal growth factor (EGF)-like domains. Each EGF-like repeat is composed of approximately 40 amino acids, and its structure is defined largely by six conserved cysteine residues that form three conserved disulfide bonds. Three of the detected changes, c.1862G>A (p.R621H), c.4028C>T (p.A1343V), and c.4492A>G (p.K1498E) are in close proximity to one of the cysteines. The alteration of the amino acid sequence might also alter the capability to create disulfide bonds and thus impair ligand binding. In addition, each EGF-like repeat can be modified by O-linked glycans at specific sites. The c.1334C>T (p.T445M) variant at such a conserved site that is predicted to undergo glycosylation might well affect protein function
2. The mutation c.4492A>G:(p.K1498E) is located in the LNR2 domain. The LNR (Lin-12/Notch repeat) region is a hallmark of the Notch receptor family and is involved in Notch signaling. An altered amino acid sequence might prevent the initiation of the ligand-induced proteolytic cleavage and release of the intracellular regulatory domain.
3. The functional consequence of the intracellularly located c.5414T>C (p.L1805P) variant is difficult to predict, however, the incorporation of a proline may impact the secondary structure of the protein.
